# Patients with IBD have a more cautious attitude towards COVID-19 vaccination

**DOI:** 10.3389/fimmu.2022.1077308

**Published:** 2023-01-18

**Authors:** Shurong Hu, Jingwen Liu, Shuyan Li, Qiangqiang Wu, Xiaoying Wang, Dingting Xu, Yan Chen

**Affiliations:** ^1^ Center of Inflammatory Bowel Disease, Department of Gastroenterology, the Second Affiliated Hospital, Zhejiang University School of Medicine, Hangzhou, China; ^2^ Department of Nursing, the Second Affiliated Hospital, Zhejiang University School of Medicine, Hangzhou, China

**Keywords:** COVID-19, vaccine, inflammatory bowel disease, potential acceptance, hesitancy

## Abstract

**Background:**

To understand the awareness of COVID-19 vaccine, the willingness to vaccinate and the influencing factors of willingness to vaccinate in inflammatory bowel disease (IBD) patients.

**Methods:**

The online questionnaire was distributed to conduct a survey to analyze and evaluate the willingness, awareness and trust in vaccines of IBD patients. Bivariate analyses and logistic regression models were used to analysis influencing factors.

**Results:**

We sent the questionnaire to the WeChat group for patient management and 304 patients responded, out of which 16 respondents had to be excluded and 288 respondents were included for the analysis. Among them, 209 patients vaccinated with COVID-19 vaccine. Among the non-vaccinated 79 patients, the main reasons for their concerns were afraid of vaccination aggravating IBD and fear of adverse effects. Our results showed that IBD patients with long disease duration were more willing to receive COVID-19 vaccination (P<0.05). We also observed that a high perception of benefits and cues to action to receive the vaccine were the two most important constructs affecting a definite intention for COVID-19 vaccination (P<0.05).

**Conclusions:**

Patients with IBD have a more cautious attitude towards COVID-19 vaccination, which may lead to a higher rate of vaccine hesitancy. Further efforts should be made to protect patients with IBD from COVID-19 infections and achieve adequate vaccination coverage.

## Introduction

By the middle of March 2022, the coronavirus-19 disease (COVID-19) had infected more than 450 million people and caused nearly 6 million death worldwide (1). COVID-19 has affected almost all age groups in both previously healthy individuals and those with chronic disease including inflammatory bowel disease (IBD) (2–4). Patients with IBD particularly in the presence of systemic corticosteroids, immunosuppressive agents, or biologics are supposed to be at moderate-to-high risk who are sensitive to COVID-19 and complications (5). Vaccination is likely to be especially important in high-risk individuals like IBD patients to suppress viral dissemination and protect individual patients from COVID-19 (6).

Previous studies showed that non-live vaccines are implicated safe in patients with IBD irrespective of IBD treatment, although patients receiving certain types of immunomodulatory at the time of vaccination may have reduced vaccine immune responses (7, 8). In fact, patients with IBD are more at risk of infectious complications than the non-IBD population once infected with COVID-19 (9). Studies have demonstrated that vaccines are also protective for patients with IBD, which follows as COVID-19 vaccination is even more essential for the protection of IBD patients (10).

However, patients with IBD have more concerns about the adverse reactions and effectiveness of vaccines than ordinary people before vaccination, which leads to vaccine hesitancy (11, 12). At present, there are still many patients with IBD in China who are reluctant to receive the COVID-19 vaccine (13, 14). Therefore, we conducted a questionnaire survey of patients with IBD to understand the attitude and potential influencing factors of IBD patients to receive COVID-19 vaccine. The study will help to enhance vaccination attitude and COVID-19 vaccination coverage among patients with IBD. The study may also help gastroenterologists educate and inform their patients on the favorable of COVID-19 vaccination, and give recommendations for further vaccination (15).

## Material and methods

### Study design

**Figure 1:**
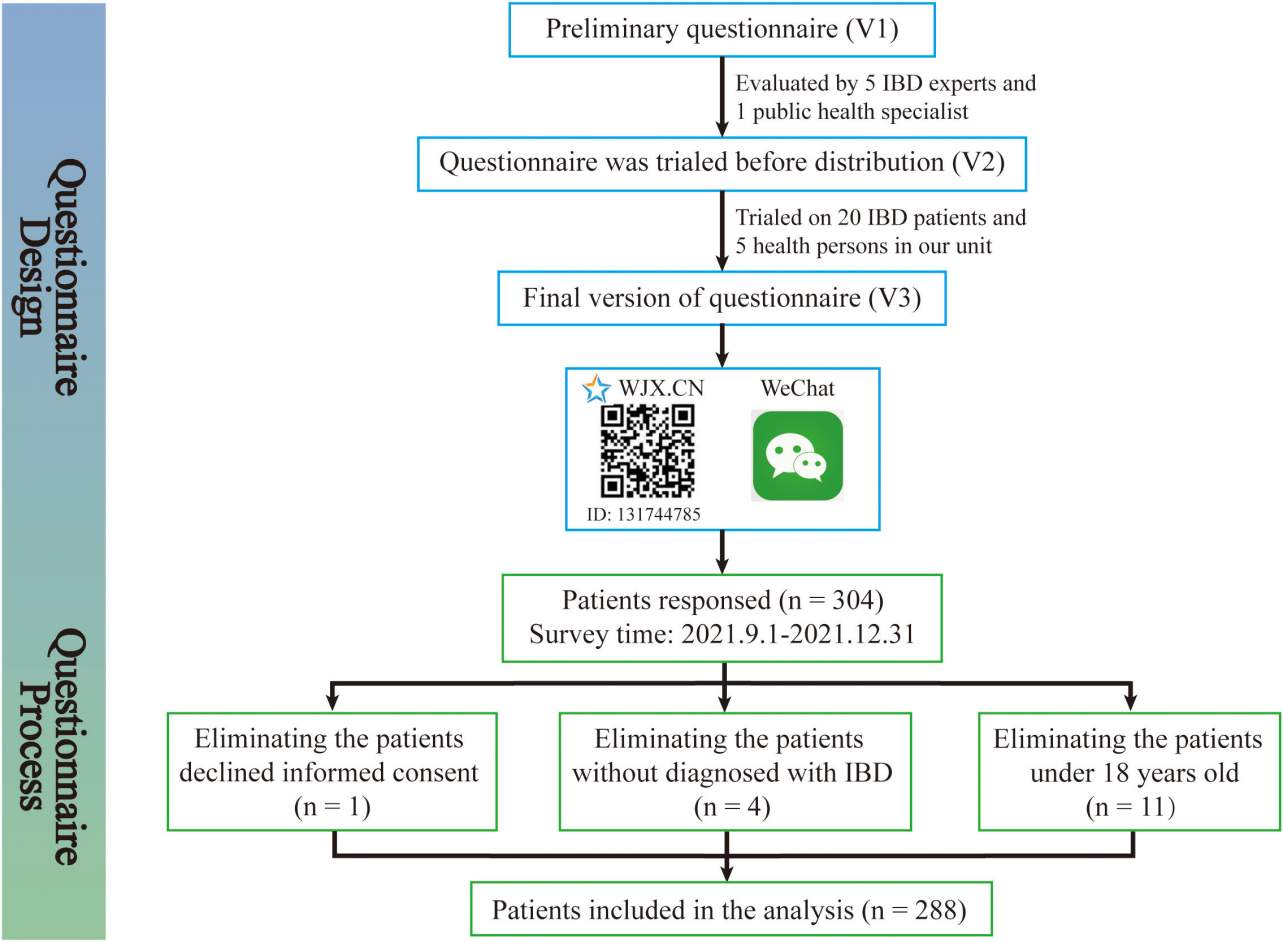
Patients enrollment flow chat. The patients enrollment flow chat was created to demonstrate the questionnaire design and questionnaire process in detail. The questionnaire was evaluated and modified by IBD experts and was produced by Wenjuanxing, which is a free and open platform for survey design. We sent the questionnaire to the WeChat group for patient management and 304 patients responded, out of which 16 respondents had to be excluded.

The study was conducted at the Second Affiliated Hospital, Zhejiang University School of Medicine (SAHZU) from September 1, 2021 to December 31, 2021. All adult IBD patients from the SAHZU Crohn’s and Colitis Center, with a confirmed diagnosis of IBD were eligible to participate in the study. Inclusion criteria included age greater than or equal to 18 years and diagnosed with Crohn’s disease or ulcerative colitis. Participants were invited to complete an online questionnaire. Patients were excluded if they had difficulty in understanding the content. As shown in **Figure 1**, the questionnaire was evaluated and modified by IBD experts and was produced by Wenjuanxing (16), which is a free and open platform for survey design. An agreement statement that the participation was voluntary was provided on the first page of the Wenjuanxing. The participation was anonymously, and the consent was obtained by clicking on the agree button. We sent the questionnaire to the WeChat group for patient management, and a total of 304 patients responded this online questionnaire, out of which 16 (5.3%) respondents had to be excluded: 1 (0.3%) declined informed consent, 4 (1.3%) were not IBD patients, and 11 (3.6%) patient was under 18 years old. Due to the web-based design of the study, the written consent form was replaced by clicking “I agree” on the information page. This study was approved by the Institutional Review Board of the Ethics Committee of the Second Affiliated Hospital, School of Medicine, Zhejiang University in China (IR2021001361).

### Sample and patients’ characteristics

The COVID-19 vaccine hesitancy rate was reported to be 18% (12–24%) among patients with IBD in different countries. This study was a cross-sectional study to evaluate the acceptance rate of COVID-19 vaccination among patients with IBD in China, requiring bilateral tests, α was 0.05. The allowable error was 0.04. Using PASS 15 software to calculate the sample size N=288 cases. Considering the 20% loss rate, at least 360 cases needed to be included in this case as the study object. The social demographic profile included current age, gender, marital status (married, not married, divorced), working condition (full-time, part time, students, unemployment, retirement), education level (primary/middle school graduate, high/secondary vocational school graduate, bachelor’s degree, master’s or doctorate’s degree) and level of income per month (under ¥2000 (US $315), ¥2000-¥5000 (US $315-$786), ¥5000-¥10000 (US $786-$1572), ¥10000-¥20000 (US $1572- $3144) and above ¥20000 (US $3144). The clinical disease profile consisted of a type of disease and disease activity, which were reported by patients with IBD themselves using the validated patient-based Harvey-Bradshaw Index (HBI) items for patients with CD and the Patient-based Simple Clinical Colitis Activity Index (P-SSCAI) for patients with UC (17, 18). HBI score>4 or P-SCCAI score ≥5 was rated as active, while HBI score ≤ 4 or P-SCCAI score <5 was rated as remission (17, 18). The health belief model (HBM) was used to measure the patients’ perception of COVID-19 and COVID-19 vaccination (19, 20). The HBM comprised 15 items, which involved perceived susceptibility, severity, benefits, barriers and cues to action (21). All the response options were “strongly agree”, “agree”, “disagree” or “strongly disagree”. The full online questionnaire is provided as **Supplemental material 1**.

### Statistical analysis

Data analyses were performed using IBM SPSS software (version 25.0, Chicago, Illinois, USA). For quantitative variables, mean and standard deviation or inter-quartile range [IQR] were calculated, and comparisons of differences between groups were compared using t-tests if normally distributed, or with the Wilcoxon test if non-normally distributed. Categorical variables were expressed as frequency or percentages, and differences in frequencies between groups were calculated using χ2 test or Fisher’s exact test. Furthermore, univariate analysis was used to determine characteristics associated with the definite intention to take the COVID-19 vaccine. Multivariate logistic regression was used to assess whether these individual variables were related to outcomes. Odds ratios (OR), 95% confidence intervals (95% CI) and p-values were calculated for each independent variable. For all tests, 2-sided p-value of 0.05 will be used for defining the level of significance.

## Results

### Characteristics of IBD patients

As shown in **Figure 1**, 304 individuals completed the questionnaire. Of those, 288 respondents with a diagnosis of IBD were included for the analysis. 166 (57.6%) were male and 122 (42.4%) were female; 251 (87.2%) had Crohn’s disease and 37 (12.8%) had ulcerative colitis; According to the HBI and the P-SCCAI scores, 75.7% (28/37) of UC patients and 96.41% (242/251) of CD patients were in clinical remission; the median age was 35 years (IQR, 25–45); 185 patients (64.2%) were married and 35.8% patients were single or divorced; Approximately 65.3% (188/288) of the patients attained higher levels of education (12 years of education or more); The employment status of the patients with IBD was also assessed in our survey. 56.9% (164/288) of the patients had full-time work, 3.5% had part-time work, 11.8% (34/288) were students, and 27.8% were unemployed or retired; In terms of medical therapy, 82 (28%) IBD patients were on 5-ASA therapy, 23 (8%) were steroids therapy, 96 (33%) were taking immunosuppressant therapy, 222 (77%) were taking biologics therapy and 105 (36%) were on enteral nutrition; More than half of respondents reported a disease duration of IBD of more than 3 years (**Table 1**).

**Table 1:** Patient characteristics (n=288).

Sex, n (%)		
Male	166	57.6%
Female	122	42.4%
Disease, n (%)		
CD	251	87.2%
CD remission	242	96.4%
CD active	9	3.6%
UC	37	12.8%
UC remission	28	75.7%
UC active	9	24.3%
Age, median (range)	35	25-45
Age group (years)		
18-25	74	25.7%
26-35	87	30.2%
36-45	62	21.5%
46-75	65	22.6%
Marital status, n (%)		
Married	185	64.2%
Non-Married	103	35.8%
Highest level of education completed, n (%)		
Primary or middle school certificate	58	20.1%
High school/Secondary vocational school	42	14.6%
Bachelor’s level	168	58.3%
Master’s and Doctorate’s level	20	7.0%
Self-reported monthly income, n (%)		
<¥2000 (< US $315)	13	4.5%
¥2000-¥5000 (US $315- $786)	75	26.0%
¥5000-¥10000 (US $786- $1572)	90	31.3%
¥10000-¥20000 (US $1572- $3144)	66	22.9%
> ¥20000 (> US $3144)	44	15.3%
Working condition, n (%)		
Full-time	164	56.9%
Part-time	10	3.5%
Students	34	11.8%
Unemployment/Retirement/others	80	27.8%
Concomitant therapy, n (%)		
Enteral nutrition	105	36.5%
Biologics therapy	222	77.0%
Ustekinumab	20	9.0%
Vedolizumab	32	14.4%
Infliximab/Adalimumab	170	76.6%
Immunosuppressants	96	33.3%
Steroids	23	8.0%
5-ASA	82	28.5%
Duration of disease, n (%)		
Less than 1 year	53	18.4%
1-3 years	76	26.4%
3-10 years	118	41.0%
More than 10 years	41	14.2%

### COVID-19 vaccine information sources and vaccination

**Figure 2:**
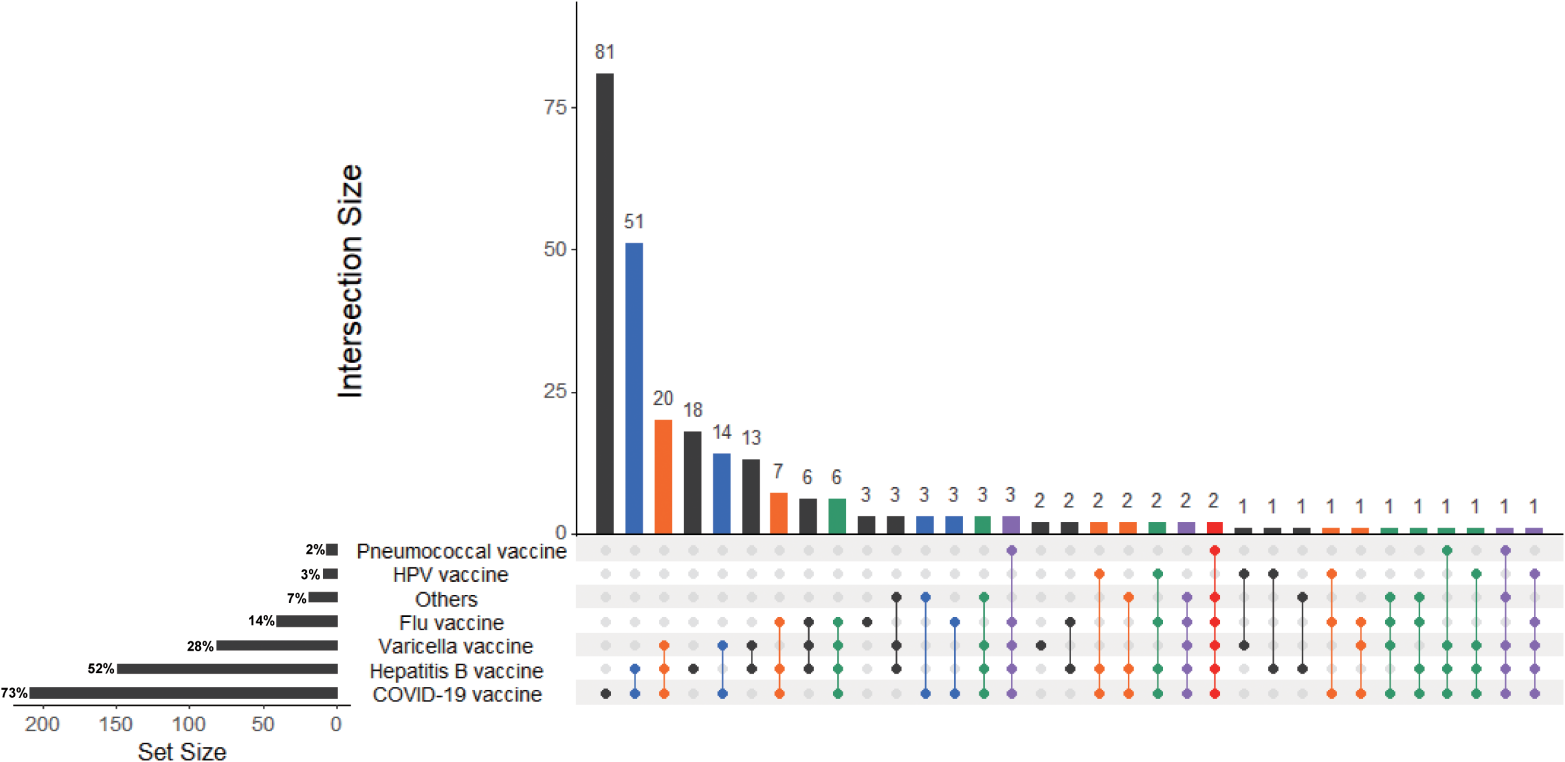
UpsetR plot of IBD patients received COVID-19 vaccines or inoculated other vaccines in the previous year. The UpSetR plot was created for IBD patients who received COVID-19 vaccines or inoculated flu vaccine, hepatitis B vaccine, pneumococcal vaccine, varicella vaccine, cervical HPV vaccine and other vaccines in the previous year. The horizontal bars with labels at the lower left of the panel represent the seven data sets of patients who received different vaccines, with the length of each bar displaying the total set size. The dot pattern to the right shows the intersections between the sets. The vertical bars at the top show the size of the corresponding intersection, ranked by decreasing set size, where a gray dot indicates an empty set and a single black dot indicates no intersection with another set.

Among 288 IBD patients, 209 patients vaccinated with COVID-19 vaccine, which were defined as vaccinated population. In addition, 79 patients did not vaccinate with COVID-19 vaccine were defined as non-vaccinated population. In addition, 81.1% (30/37) patients with UC and 71.3% (179/251) patients with CD vaccinated with COVID-19 vaccine. Furthermore, almost half of patients 149 (52%) inoculated hepatitis B vaccine, less than one third (28%) patients inoculated varicella vaccine, 14% patients inoculated flu vaccine. There were 81 patients who received COVID-19 vaccine but had never been inoculated with other vaccines before, which is much more than those who had cross-vaccination (**Figure 2**).

Among the non-vaccinated patients, the main reasons for their concerns were as follows: afraid of vaccination aggravating IBD (39%), fear of adverse effects (22%), worry about the current use of drugs affecting the efficacy of vaccination (14%), unknow the most appropriate time to vaccinate (8%), other reasons (17%) (**Table 2**).

**Table 2:** Main rejecting reasons of the COVID-19 vaccine in the refusals.

	Patients (79)
Afraid of vaccination aggravating IBD	31 (39.2%)
Fear of adverse effects	18 (22.8%)
Worry about the current drugs affecting the efficacy of vaccination	11 (13.9%)
Unknowing the most appropriate time to vaccinate	7 (8.9%)
Other reasons	12 (15.2%)

The crude OR analysis of sociodemographic, clinical disease profile and perception about COVID-19 vaccine in general were shown in **Table 3**. The results showed that IBD patients with long disease duration were more willing to receive COVID-19 vaccination (P<0.05) (**Table 3**). There were no significant differences between the vaccinated population and non-vaccinated population in terms of gender, disease type, disease activity, other vaccinations, access to vaccine information, consultation with medical staff about vaccines, and understanding of preventive measures before vaccination (P>0.05).

**Table 3:** Factors on socioeconomic characteristics (univariate analysis).

	Vaccinated(n=209)	Non-vaccinated(n=79)	p-value
**Gender**			0.498
female	86(70.49%)	36(29.51%)	
male	123(74.10%))	43(25.90%)	
**Current age**	35(26-44)	35(25-48)	0.222
**Marital status**			0.730
married	133(71.89%)	52(28.11%)	
non-married	76(73.79%)	27(26.21%)	
**Working conditions**			0.202
Full-time/part time work	131(75.29%)	43(24.71%)	
Students/unemployed/retired	78(68.42%)	36(31.58%)	
**Self-reported monthly income**			0.555
Under 5000 ¥/5000-10000¥	127(71.35%)	51(28.65%)	
10000-20000¥/above 20000¥	82(74.55%)	28(25.45%)	
**Disease type**			0.214
CD	179(71.31%)	72(28.69%)	
UC	30(81.08%)	7(18.92%)	
**Disease duration**			**0.013**
<3years	103(79.84%)	26(20.16%)	
≥3years	106(66.67%)	53(33.33%)	
**Disease activity**			0.609
active	14(77.78%)	4(22.22%)	
stable	195(72.22%)	75(27.78%)	
**Other vaccinations**			0.750
Yes	128(71.91%)	50(28.09%)	
No	81(73.64%)	29(26.36%)	

Bold values indicates P value<0.05 which had statistical significance.

### Attitudes and health belief of COVID-19 vaccine

In the univariate analysis, most of the constructs in the HBM model (22) were obviously related with having a definite intention for COVID-19 vaccination (**Table 4**). Perception that the possible side-effects of COVID-19 vaccination would interfere with usual activities under the perceived benefit construct (OR = 2.304, 95% CI 1.393–3.817) was the strongest predictor for a definite intention. Under the cues to action construct, perception that patients would only take the COVID-19 vaccine if they were given adequate information were associated with having a higher definite intention to vaccinate (OR = 3.789, 95% CI 2.043–7.027) (**Table 5**). In the multivariate logistic regression analysis, the patients with IBD over 3 years were correlated with having a higher definite intention to vaccinate (OR = 2.217, 95% CI 1.197–4.016) (**Table 5**).

**Table 4:** Factors on HBM model (univariate analysis).

	Vaccinated(n=209)	Non-vaccinated(n=79)	p-value
Perceived susceptibility			
Great chance of getting COVID-19 in next months			0.739
Strongly agree/agree	21(70.0%)	9(30.0%)	
Disagree/strongly disagree	188(72.9%)	70(27.1%)	
Worry about the likelihood of getting COVID-19			0.781
Strongly agree/agree	59(73.8%)	21(26.2%)	
Disagree/strongly disagree	150(72.1%)	58(27.9%)	
Getting COVID-19 is currently a possibility for me			0.853
Strongly agree/agree	7(70.0%)	3(30.0%)	
Disagree/strongly disagree	202(72.7%)	76(27.3%)	
Perceived severity			
Complications from COVID-19 are serious			**0.04**
Strongly agree/agree	147(69.3%)	65(30.7%)	
Disagree/strongly disagree	62(81.6%)	14(18.4%)	
I will be very sick if I get COVID-19			0.37
Strongly agree/agree	13(70.8%)	54(29.2%)	
Disagree/strongly disagree	78(75.7%)	25(24.3%)	
I am afraid of getting COVID-19			0.411
Strongly agree/agree	132(71.0%)	54(29.0%)	
Disagree/strongly disagree	77(75.5%)	25(24.5%)	
Perceived benefits			
Vaccination is a good idea because it makes me feel less worried about catching COVID-19			0.103
Strongly agree/agree	193(73.9%)	68(26.1%)	
Disagree/strongly disagree	16(59.3%)	11(40.7%)	
Vaccination decreases my chance of getting COVID-19 or its complications			0.725
Strongly agree/agree	198(72.8%)	74(27.2%)	
Disagree/strongly disagree	11(68.8%)	5(31.2%)	
Perceived barriers			
Worry the possible side-effects of COVID-19 vaccination would interfere with my usual activities			**0.0001**
Strongly agree/agree	58(52.7%)	52(47.3%)	
Disagree/strongly disagree	151(84.8%)	27(15.2%)	
Concern about the efficacy of the vaccination			**0.0001**
Strongly agree/agree	84(60.0%)	56(40.0%)	
Disagree/strongly disagree	125(84.5%)	23(15.5%)	
Concern about the safety of the vaccination			**0.045**
Strongly agree/agree	181(70.7%)	75(29.3%)	
Disagree/strongly disagree	28(87.5%)	4(12.5%)	
Concern of my affordability of getting vaccination			**0.0001**
Strongly agree/agree	61(59.8%)	41(40.2%)	
Disagree/strongly disagree	148(79.6%)	38(20.4%)	
Concern if the faulty/fake COVID-19 vaccine			0.064
Strongly agree/agree	56(65.1%)	30(34.9%)	
Disagree/strongly disagree	153(75.7%)	49(24.3%)	
Cues to action			
I will only take the COVID-19 vaccine if I was given adequate information about it			**0.007**
Strongly agree/agree	206(73.8%)	73(26.2%)	
Disagree/strongly disagree	3(33.3%)	6(66.7%)	
I will only take the COVID-19 vaccine if the vaccine is taken by many in the public			0.635
Strongly agree/agree	164(71.9%)	64(28.1%)	
Disagree/strongly disagree	45(75.0%)	15(25.0%)	

Bold values indicates P value<0.05 which had statistical significance.

**Table 5:** Factors on intention to take the COVID-19 vaccine (multivariate analysis).

	OR (95% CI)	p-value
Disease type		
CD	Reference	
UC	0.459(0.158-1.329)	0.151
Disease duration		
<3years	Reference	
≥3years	2.217(1.197-4.016)	**0.011**
Disease activity		
active	Reference	
stable	1.436(0.405-5.095)	0.575
Complications from COVID-19 are serious		
Strongly agree/agree	Reference	
Disagree/strongly disagree	0.954(0.647-1.407)	0.813
Worry the possible side-effects of COVID-19 vaccination would interfere with my usual activities		
Strongly agree/agree	2.304(1.393-3.817)	**0.001**
Disagree/strongly disagree	Reference	
Concern about the efficacy of the COVID-19 vaccination		
Strongly agree/agree	Reference	
Disagree/strongly disagree	0.765(0.450-1.298)	0.32
Concern about the safety of the COVID-19 vaccination		
Strongly agree/agree	Reference	
Disagree/strongly disagree	0.619(0.344-1.112)	0.109
Concern of my affordability of getting the vaccination		
Strongly agree/agree	Reference	
Disagree/strongly disagree	1.091(0.723-1.648)	0.678
I will only take the COVID-19 vaccine if I was given adequate information about it		
Strongly agree/agree	3.789(2.043-7.027)	**<0.0001**
Disagree/strongly disagree	Reference	

Bold values indicates P value<0.05 which had statistical significance.

## Discussion

The present study examined the vaccinated and non-vaccinated patients with IBD, and investigated various sociodemographic, health-related and behavioral predictors for these patients based on the HBM model. The acceptance of the COVID-19 vaccine in patients with IBD has not been adequately evaluated, and the reasons for the hesitancy of patients with IBD about the COVID-19 vaccine are unknown. A total of 288 IBD patients were finally included in this study, of which 72.6% had received the COVID-19 vaccine, which was consistent with other findings in Italy and United States (23, 24). We found that 27.4% of IBD patients were hesitant to vaccinate against COVID-19, with significantly associated factors in the adjusted analysis being disease duration, perceived benefit and cues to action.

It was reasonable to find higher intention of vaccination among respondents in the long disease duration, as their more stable condition, a better understanding of the vaccination, or a greater willingness to follow gastroenterologist advice (25). With regard to sociodemographic and health-related predictors, none were found to be significant in terms of the intention to receive COVID-19 vaccines. These included demographic considerations, such as personal status, socio-economic level and health-related factors, such as perceived health status, disease type, disease activity or having been vaccinated with other vaccines like flu vaccine in the previous year.

Previous studies have shown that inactivated vaccines such as hepatitis B vaccine, varicella vaccine and flu vaccine are well tolerated and safety (26, 27). Meanwhile, the guidelines for the vaccination of patients with autoimmune diseases or immunosuppressive therapy suggest that inactivated vaccines can benefit the patients without interruption of treatment (6, 28). In China, the COVID-19 vaccines are all inactivated vaccines, so it is feasible for patients with IBD to receive COVID-19 vaccines. In our study, the most common reasons for COVID-19 vaccine hesitancy were afraid of vaccination aggravating IBD, fear of adverse effects and worry about the current use of drugs affecting the efficacy of vaccination. These suggested that patients with IBD would be more cautious before choosing vaccination. In addition, those who were willing to be vaccinated in this study were more concerned about the effectiveness of the vaccine. While patients who refused to be vaccinated might have other immune diseases, and they were afraid that vaccination might aggravate existing conditions. These results suggested that the vaccinated group may overestimate the benefits of vaccination, while the non-vaccinated group may be unaware of the vaccine.

Whether patients with IBD should be vaccinated against COVID-19, D’Amico et al. suggested that they should receive COVID-19 vaccines, regardless of medication or comorbidities (10). According to the international consensus, it holds that no matter what drug treatment IBD patients receive, inactivated vaccines are safe for them, and vaccination is not associated with the onset or worsening of IBD (6). However, the inclusion criteria of vaccines that are currently on the market do not include patients with immune diseases or receiving immunosuppressive therapy (29, 30). Therefore, patients with IBD still need to be cautious when vaccinating against COVID-19.

In our study, multivariable analysis found that HBM constructs were associated with vaccination intention, which was in concordance with other studies (31). Especially, the findings of our study observed that a high perception of benefits and cues to action to receive the vaccine were the two most important constructs affecting a definite intention for COVID-19 vaccination. In our study, the perceived barriers against COVID-19 immunizations, especially worried about side effects had been also reported in other studies related to the introduction of a new vaccine (32). The findings implied that it should be valued the established safety and efficacy before the new vaccine made available to the public. However, perception of susceptibility was not a significant predictor in the study. While cues to actions were found to be important, namely patients would only take the COVID-19 vaccine if they were given adequate information. Therefore, it is important to provide the public with adequate information, especially strong evidence from field trials of vaccine safety and efficacy. These findings suggested that the promotion of COVID-19 vaccination in the form of advertisements and recommendations may serve as cue to action for vaccination.

There were several limitations to our study. Firstly, the present study was self-reported from patients and certainly related to the retrospective nature which might have been recalling bias and selection bias. Secondly, our study focused on the IBD patients, we just compared the factors among IBD patients and didn’t compare the factors with non-IBD controls. Thirdly, detailed characteristics of individual patients were not available. Other limitations to mention were that the survey respondents represented only a small proportion of all Chinese IBD patients and thus may not be generalizable to all Chinese IBD patients or other countries, especially considering differences in rates of both COVID-19 and IBD. The strength of our study was that we did a comprehensive survey on sociodemographic, clinical disease profile and health belief in IBD patients.

COVID-19 vaccination for the general population has been proceeding in China. Meanwhile, IBD specialists are still called upon in conducting a campaign to convince their patients to get vaccinated. Our study suggests that patients with IBD are more cautious about vaccinating against COVID-19, resulting in a higher rate of vaccine hesitancy. It is necessary to explain to patients that the benefits of vaccines outweigh the risks, especially in patients without immune disease. Further efforts should be made to protect patients with IBD from COVID-19 infections and achieve adequate vaccination coverage. Healthy education and advice from government and IBD experts may facilitate COVID-19 vaccination.

## Data availability statement

The original contributions presented in the study are included in the article/**Supplementary Material**. Further inquiries can be directed to the corresponding author.

## Ethics statement

The studies involving human participants were reviewed and approved by the Institutional Review Board of the Ethics Committee of the Second Affiliated Hospital, School of Medicine, Zhejiang University in China (IR2021001361). The patients/participants provided their written informed consent to participate in this study.

## Author contributions

YC, SH and JL participated in study design and study conception. SL collected the questionnaire data. SH, JL, performed statistical analysis. QW, XW, and DX performed the check of results. SH and JL drafted the manuscript. All authors provided critical review of the manuscript and approved the final draft for publication. All authors contributed to the article and approved the submitted version.
