# Supplementary material for: Patients with IBD have a more cautious attitude towards COVID-19 vaccination
Source: Front Immunol. 2023 Jan 18;13:1077308. doi: 10.3389/fimmu.2022.1077308 (PMC9889360; doi:10.3389/fimmu.2022.1077308)
Supplement: Supplementary file 1 [file Table_1.docx]

Supplementary Material

# Patient online questionnaire

1.1. What is your disease type?

Crohn's disease / Ulcerative colitis / Atypical enteritis / Not the above

1.2. How long have you been diagnosed with inflammatory bowel disease (IBD, mainly including Crohn's disease and ulcerative colitis): [single choice]

a) < 1 year

b) 1 ~ 3 years (excluding 3 years)

c) 3 ~ 10 years (excluding 10 years)

d) ≥ 10 years

1.3. Your gender: Male / female

1.4. Your age:

a）<18

b）18～24

c）25~54

d）55-64

e）>65

1.5. Your current working status:

Full time work / intermittent work / retirement / student / unemployed / other

1.6. Your occupation (the first three answers to the above question indicate that you have a job):

Staff from government, Party organizations, enterprises and institutions / professional technicians / clerks and relevant personnel / personnel in commerce and service industry / production personnel in agriculture, forestry, animal husbandry, fishery and water conservancy / operators of production and transportation equipment and relevant personnel / soldiers

1.7. Degree of education:

Junior high school or below / senior high school or technical secondary school / junior college or undergraduate / graduate student or above

1.8. Your current religious belief:

No religion / Buddhism / Christianity / other religions

1.9. Your nationality:

Han / other nationalities

1.10. Your marital status: [single choice]

a） unmarried

b） married

c） divorce

d） Widowed

e） other

11. Your average monthly income is: [single choice]

a）< 2000

b） 2000 ~ 5000 (excluding 5000)

c） 50 ~ 10000 (excluding 10000)

d） 10000 ~ 20000 (excluding 20000)

e） ≥ 20000

12. Which of the following drugs have you used in the past six months (multiple choices are allowed):

a) Mesalazine (such as Pentasa / Etiasa / Salofalk / ANJISHA / Sulfasalazine, etc.) (All oral, enema and suppository belong to this category)

c) Glucocorticoids (e.g. Prednisone / Methylprednisolone)

d)Immunosuppressants such as azathioprine (such as Imuran) or methotrexate (intramuscular or oral)

f) thalidomide

g) Enteral nutrition (such as Ensure or fresubin, Peptisorb, TPF, etc.)

h) Anti TNF biological agents (like IFX or ADA)

i) Anti integrin biological agents (VDZ)

j) Anti IL-23 biological agents (UST)

k) Other drugs: Not sure

13. (Patients with UC) how many times did you use the bathroom on average every day (24 hours) in the previous week?

a) 0-3 times, b) 4-6 times, c) 7-9 times, d) more than 9 times

14. (patients with UC) how many times did you get up and use the bathroom each night in the previous week?

a) Never, b) 1-3 times, c) more than 3 times

15. (Patients with UC) In the previous week, could you hold enough time when you wanted to use the bathroom?

a) Totally, b) not really, c) immediately, d) fecal incontinence

16. (Patients with UC) how many times did you find blood in the stool in the previous week?

a) Never, b) less than half, c) almost half, d) more than half

17. (Patients with UC) On a scale of 1 to 10,how is your overall health status in the last week? (1 = very poor, 10 = perfect)?

a) Good ( ≥ 7); b) Better(6); c) Difference(5); d) Very poor(4); e) Very bad(< 4)

18. (Patients with UC) Were there any other parenteral complications in the last week? (multiple choices are allowed)

a) Arthritis, b) uveitis, c) nodular erythema, d) gangrenous pyoderma

19. (Patients with CD) What was your general condition the day before?

a) 0 = good, b) 1 = slightly poor, c) 2 = poor, d) 3 =very poor, e) 4 = bad

20. (Patients with CD) how was your abdominal pain the day before?

a) 0 = none, b) 1 = mild, c) 2 = medium, d) 3 = severe

21. (Patients with CD) How many times do you have loose stool the day before?

a) 0-1, b) 2-3, c) 4-5, d) 6-7, e) 8-9, f) 10 or more

22. (Patient with CD) Do you have abdominal mass?

a) 0 = none, b) 1 = suspicious, c) 2 =diagnosis, d) 3 = with tenderness

23. (Patient with CD) Do you have the following complications

a) Arthritis, b) iritis, c) nodular erythema, d) pyoderma gangrenosum, e) aphthous ulcer, f) anal fissure, g) new anal fistula, H) anal abscess

Part II: vaccine information sources

24. Have you discussed the 2019-nCoV vaccine with your doctor?

a) Yes b) no

25. (Patients choose a from question 24 to answer) who is the doctor discussed the 2019-nCoV vaccine with you?

a) IBD exclusive doctor (refers to the doctor who is more professional in the diagnosis and treatment of your ulcerative colitis / Crohn's disease, such as the professional doctor who opens IBD specialist clinic or has many IBD patients)

b) Non IBD exclusive doctor

26.(Patients choose a from question 24 to answer) Does your doctor recommend you inject the 2019-nCoV vaccine?

a) The doctor supported 2019-nCoV vaccine injection

b) The doctor objected to 2019-nCoV vaccination

c) The doctor doesn't know if I can get the 2019-nCoV vaccine

27. Have you read or heard anything about the vaccination knowledge of 2019-nCoV vaccine for IBD patients? Yes / no

If yes, the main source of knowledge?

a. network (such as official website, etc.)

b. doctor/nurse

c. patients.

28. Does the vaccination knowledge strengthen your confidence in injecting the 2019-nCoV vaccine?

a) Yes,

b) No, I don't dare to inject after knowing information

c) I still don't know if I can inject it

29. Did you start vaccination because of the vaccination knowledge?

a) Yes

b) NO

c) I'm ready, but I haven't started the injection yet

Patients answers with a of 27th question continue to answer below questions:

What are the main network sources of your 2019-nCoV vaccine (within 3 answers, multiple choices are allowed)

a) WeChat official account

b) Bilibili

c) Microblog

d) tiktok

e) Little red book

f) Apps

g) IBD knowledge website

h) Other network sources

28. Patients answers with a of last question (WeChat official account), are you a subscriber of WeChat official account of CCCF(the China Crohn’s & Colitis Foundation)?

a) Yes b) no

29. Are you a volunteer of CCCF?

a) Yes b) no

Part III: vaccine implementation

33. Have you received the 2019-nCoV vaccine?

a) Yes, b) no

34. Choose a from question 33 to answer, is it convenient for you to receive vaccination?

a) Yes, b) no

35. Choose a from question 33 to answer, have you completed the 2019-nCoV vaccination?

a) Yes, b) no, still need 1-2 injection

36. Choose a from question 33 to answer, which vaccine manufacturer did you vaccinate?

a) Sinopharm Wuhan institute of biological products CO.,LTD

b) Sinopharm Beijing institute of biological products CO.,LTD

c) Sinovac BiotechCO., LTD

d) Academy of Military Medical Sciences

e) Anhui Zhifei Longcom Biopharmaceutical Co.,Ltd

f) Not clear

F) Other___

37. Choose a from question 33 to answer,why did you not get the 2019-nCoVvaccine?

a) I don't want to be vaccinated

b) I wanted to be vaccinated, but because I was an IBD patient, the vaccination center did not recommend me to be vaccinated

c) I wanted to be vaccinated, but because I was an IBD patient, the vaccination center refused to let me be vaccinated

38. Choose b from question 33 to answer, what are your main concerns about not getting the 2019-nCoV vaccine?

a) I'm worried that the 2019-nCoVvaccine will aggravate IBD

b) I am worried that the current use of drugs will affect the efficacy of the new crown vaccine

c) I don't know which vaccine to choose

d) I don't know when to vaccinate is the safest

e) I am worried about the adverse reactions after vaccination, such as fever, fatigue, headache, bleeding, abdominal pain, thrombosis and so on

f) Other reasons

39. have you ever been vaccinated with other IBD related vaccines (such as influenza, hepatitis B vaccine, chicken pox, etc.) yes or no?

Select Yes to continue below:

a) influenza vaccine, b) hepatitis B vaccine, c) pneumococcal vaccine, d) varicella vaccine, e) cervical HPV vaccine, f) Other vaccines

Part IV: vaccine belief

40. If there is evidence that the vaccine is very safe and effective for IBD patients, are you willing to vaccinate?

a) Willing to vaccinate b) unwilling to vaccinate

41. I have a hunch that my chances of infecting COVID-19 will be great in the next few months.

a) Strongly agree, b) agree, d) disagree, e) strongly disagree

42. I am very worried that I will infect COVID-19.

a) Strongly agree, b) agree, d) disagree, e) strongly disagree

43. I feel that I may have been infected with COVID-19.

a) Strongly agree, b) agree, d) disagree, e) strongly disagree

44. I think the complications of COVID-19 are very serious.

a) Strongly agree, b) agree, d) disagree, e) strongly disagree

45. I feel that if I am infected with COVID-19, I will be very sick.

a) Strongly agree, b) agree, d) disagree, e) strongly disagree

46. I am very afraid that I will infect COVID-19.

a) Strongly agree, b) agree, d) disagree, e) strongly disagree

47. I think vaccination is a good idea because it makes me less worried about COVID-19 infection.

a) Strongly agree, b) agree, d) disagree, e) strongly disagree

48. I think vaccination will reduce my chances of contracting COVID-19 or its complications.

a) Strongly agree, b) agree, d) disagree, e) strongly disagree

49. I am worried that the new crown vaccine will have side effects and interfere with my daily activities

a) Strongly agree, b) agree, d) disagree, e) strongly disagree

50. I'm worried about the vaccination effect of the vaccine

a) Strongly agree, b) agree, d) disagree, e) strongly disagree

51. I am very concerned about the safety of vaccination

a) Strongly agree, b) agree, d) disagree, e) strongly disagree

52. I am worried that the vaccination of the vaccine will increase my financial burden (for example, in case of complications)

a) Strongly agree, b) agree, d) disagree, e) strongly disagree

53. I am worried about whether there will be defective or counterfeit vaccines

a) Strongly agree, b) agree, d) disagree, e) strongly disagree

54. If I get enough information, I will get the new crown vaccine

a) Strongly agree, b) agree, d) disagree, e) strongly disagree

55. I will only vaccinate when many people are vaccinated with the vaccine

a) Strongly agree, b) agree, d) disagree, e) strongly disagree
